# Adsorptive Desulfurization of Model Gasoline by Using Different Zn Sources Exchanged NaY Zeolites

**DOI:** 10.3390/molecules22020305

**Published:** 2017-02-17

**Authors:** Jingwei Rui, Fei Liu, Rijie Wang, Yanfei Lu, Xiaoxia Yang

**Affiliations:** Tianjin Key Laboratory of Applied Catalysis Science and Technology, Department of Catalysis Science and Engineering, School of Chemical Engineering and Technology, Tianjin University, Tianjin 300354, China; rjw19920112@163.com (J.R.); 15022364970@163.com (F.L.); rijiewang@163.com (R.W.); luyanfei19870825@163.com (Y.L.)

**Keywords:** Zeolite, Zinc sources, adsorption, desulfurization, ion exchange

## Abstract

A series of Zn-modified NaY zeolites were prepared by the liquid-phase ion-exchange method with different Zn sources, including Zn(NO_3_)_2_, Zn(Ac)_2_ and ZnSO_4_. The samples were tested as adsorbents for removing an organic sulfur compound from a model gasoline fuel containing 1000 ppmw sulfur. Zn(Ac)_2_-Y exhibited the best performance for the desulfurization of gasoline at ambient conditions. Combined with the adsorbents’ characterization results, the higher adsorption capacity of Zn(Ac)_2_-Y is associated with a higher ion-exchange degree. Further, the results demonstrated that the addition of 5 wt % toluene or 1-hexene to the diluted thiophene (TP) solution in cyclohexane caused a large decrease in the removal of TP from the model gasoline fuel. This provides evidence about the competition through the π-complexation between TP and toluene for adsorption on the active sites. The acid-catalyzed alkylation by 1-hexene of TP and the generated complex mixture of bulky alkylthiophenes would adsorb on the surface active sites of the adsorbent and block the pores. The regenerated Zn(Ac)_2_-Y adsorbent afforded 84.42% and 66.10% of the initial adsorption capacity after the first two regeneration cycles.

## 1. Introduction

The sulfur compounds of the exhaust emissions from gasoline and diesel fuels are the main impurities in air pollutants, which are very harmful to the environment and human health [1,2]. Moreover, sulfur components poison catalysts in the exhaust gas converter for reducing CO and NO_x_ emissions [3,4]. The desulfurization of transportation fuels such as diesel and gasoline is also important from the point of application in fuel cells, as liquid hydrocarbons such as gasoline can be used conveniently as a feed in the fuel cells [5]. The fuel cells, however, require more stringent conditions on sulfur levels which are of the order of 1 ppmw, and preferably 0.1–0.2 ppmw, to avoid poisoning of the catalyst due to sulfur [6,7]. Therefore, sulfur contents in transportation fuels should be limited to a very low level with a stringent fuel specification [8,9]. According to the recently announced Euro V norms, the fuel sulfur contents will have to be reduced to as low as 10 ppmw in the near future [1].

Various methods to remove the sulfur in oil distillates have been proposed in many developed countries, such as hydro desulfurization (HDS) [10,11], adsorptive desulfurization (ADS) [12,13], oxidative desulfurization [14,15], biological desulfurization [16,17], etc. The traditional HDS process is widely employed on an industrial scale. Typically, the HDS technology is usually operated at a temperature in the range of 300–450 °C, and at a H_2_ pressure of 3.0–5.0 MPa, with CoMo/Al_2_O_3_ or NiMo/Al_2_O_3_ catalysts [18,19]. Industrially, the HDS process involves catalytic treatment with H_2_ to convert the various sulfur compounds to hydrogen sulfide and requires severe operating conditions [1]. The olefins in feedstock will react with H_2_ to form alkanes under such conditions, resulting in significant loss of the octane number [20]. Moreover, some sulfides, especially aromatic thiophenes and TP derivatives, are hard to remove by HDS [12]. In order to achieve deep desulfurization, there is a need to enlarge the reactor size and consume more energy [21]. Therefore, to overcome these drawbacks, significant efforts have been shifted to ADS. ADS is considered a promising approach with several advantages, including ambient operating conditions and selective removal of refractory thiophenic compounds.

To develop a proper adsorbent, many studies have been performed using metal oxides [22,23], carbon-based materials [24,25,26] as well as zeolites and their metal-loaded derivatives [27,28]. Among these materials, Y-type zeolites have been investigated widely due to their high surface area, size-selective adsorption capacity, high ion-exchange capacity, and good thermal stabilities [29,30]. Yang and his co-workers [12,13,28,31,32] explored Ag-, Cu-, Ni-, and Zn-exchanged Y zeolites, which exhibited a high sulfur adsorption capacity for thiophenic compounds. They proposed the π-complexation interaction mechanism of thiophenic compounds with metal-exchanged zeolites. In addition, they also noted that the adsorption performance of the Cu(I)-Y zeolites would decrease when aromatics were present in the fuel, and the decline may be attributed to the competitive adsorption of sulfur compounds and aromatics via π-complexation. Velu et al. [33] reported that ion-exchanged NH_4_-Y zeolites with transition metals such as Cu, Ni, Zn, Pd, and Ce exhibited selective adsorption of organic sulfides. Ce-exchanged NH_4_-Y zeolites exhibited a higher selectivity for the adsorption of sulfur compounds compared to the selectivity of aromatics than the other ion-exchanged zeolites. They indicated the removal of the sulfur compounds by a direct sulfur-metal (S-M) interaction rather than by π-complexation. M. Oliveira et al. The authors of [34] investigated the adsorption capacity of TP and toluene on NaY zeolites exchanged with transition metals (5 wt % Ni, Zn and Ag) and their competitive adsorption behavior systematically. The obtained results indicated the importance of inserting transitions metals in the zeolites’ structure to enhance the adsorption of both aromatic and sulfur compounds in organic liquid mixtures. Zhang, Z.Y. [35] synthesized Cu-, Zn-, Ag-exchanged and binary metal–exchanged NaY adsorbents with excellent performances and proposed a synergistic effect between different metal cations. Though progress has been made, it is still a challenge to easily synthesize such a NaY-based adsorbent with a good performance.

Zinc was also the main component of a Ni/ZnO sorbent for the adsorptive HDS of kerosene for fuel-cell applications, where ZnO in this system acts as an acceptor of sulfur that is released during the regeneration of the sulfided nickel surface species [36]. Several Zn-based adsorbents, pure metal oxide (ZnO) and mixed oxides (zinc ferrite and zinc titanate) have been attractive for gas-phase high-temperature desulfurization because of their favorable sulfidation thermodynamics, high H_2_S removal efficiency, good sulfur-loading capacity, high regenerability, and sufficient strength [37,38]. In addition, zinc-based nanocrystalline aluminum oxide has been used in the application of adsorptive desulfurization of transportation fuels [39].

Zn^2+^ has the electronic configuration 3d^10^4s^0^. It indicates that there is some donation of the electron charge transfer from the π orbital of TP to the vacant *s* orbital of Zn^2+^, simultaneously with the back-donation of electron charge transfer from the *d* orbitals of Zn^2+^ to the π* orbital of TP. Therefore, ZnY can adsorb TP. Many people have studied the adsorption desulfurization performance of ZnY zeolites. Most of them use Zn(NO_3_)_2_ as the Zn source for the ion exchange. We believe that the use of different sources of Zn can obtain different adsorption desulfurization properties. At present, very few people are engaged in this research. So in this work, cyclohexane and TP were used as a model fuel and model sulfur compound, respectively. A series of Zn-modified NaY zeolites were prepared by a liquid-phase ion-exchange method with different Zn sources and tested by adsorption of TP from model gasoline fuels. The main propose of this study was to examine and compare the desulfurization performance of ion-exchanged NaY zeolites from different Zn sources. Moreover, to uncover the adsorption mechanism, toluene and 1-hexene were added to the solution to test the selectivity. Finally, the regeneration ability and reuse of the spent adsorbents was investigated. The model gasoline fuels tested in this work used gas chromatography-flame ionization detector (GC-FID).

## 2. Results and Discussion

### 2.1. Characterization Results

**Figure 1:**
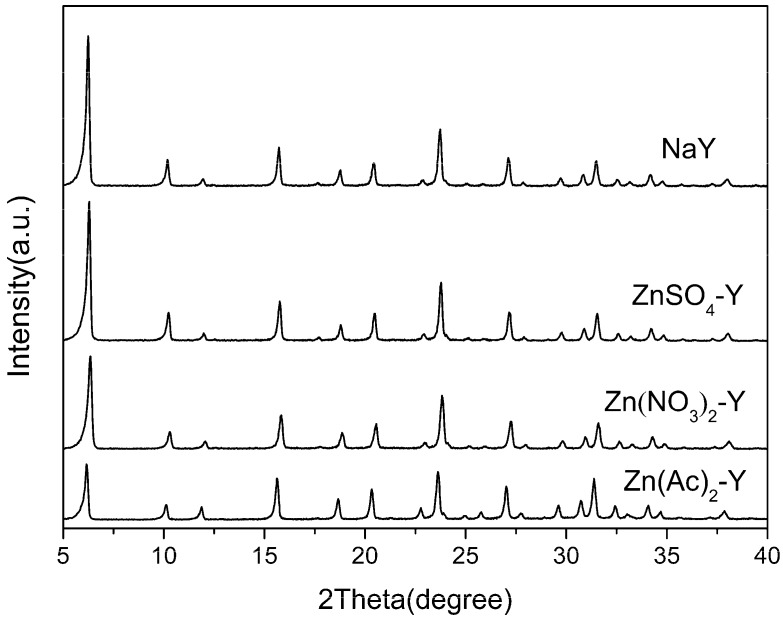
XRD patterns of different adsorbents.

XRD analysis was performed to check the crystallinity of the ZnY zeolite adsorbents and to detect the formation of metal oxides after the liquid-phase ion exchange. As illustrated in Figure 1, the powder XRD spectra of all samples exhibited a typical FAU (faujasite) framework and no significant diffraction lines associated with Zn-related compounds were identified. It is evident that the modified samples kept the original framework structure of the NaY zeolites and no new phases emerged. The results confirmed that the structures of the zeolites were not disturbed during preparation. However, their relative crystallinity decreased slightly after calcination. The relative crystallinity of Zn(Ac)_2_-Y, Zn(NO_3_)_2_-Y and ZnSO_4_-Y (10 h, 60 °C) dropped to 84.5%, 85.0% and 90.1%, respectively, which can be attributed to the crystal lattice collapse that occurred during the calcination process [40].

The chemical compositions determined by ICP-AES are summarized in Table 1. It can be observed that the ratio of the Si/Al of NaY zeolite was 2.341. The Si/Al ratios of the Zn(NO_3_)_2_-Y, Zn(Ac)_2_-Y and ZnSO_4_-Y zeolites were close to the parent NaY, suggesting that the dissolution of Al^3+^ rarely occurs during the exchange process. For the samples of Zn(NO_3_)_2_-Y, Zn(Ac)_2_-Y and ZnSO_4_-Y (10 h, 60 °C), the Na exchange degrees were 56.70%, 59.47%, 43.17%, respectively. Upon ion exchange, Zn(Ac)_2_-Y (10 h, 60 °C) exhibited the highest degree, which means it has the largest amount of adsorptive active sites. As the time went on, the ion exchange degree increased simultaneously.

**Figure 2:**
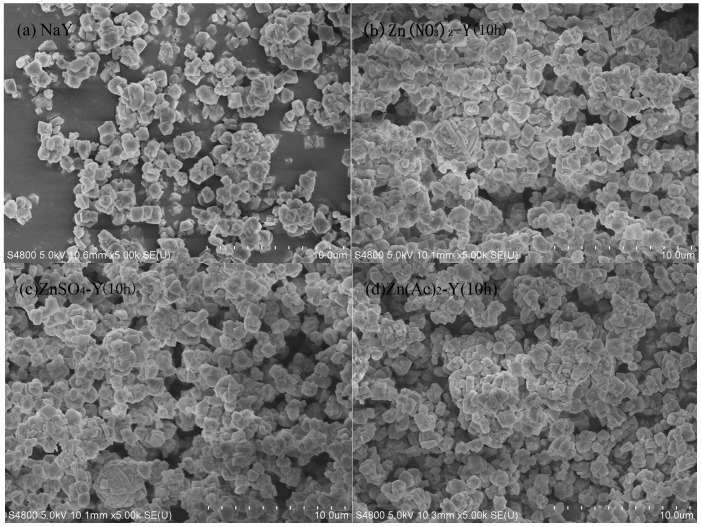
SEM images of adsorbents: (**a**) NaY; (**b**) Zn(NO_3_)_2_-Y (10 h); (**c**) ZnSO_4_-Y (10 h); (**d**) Zn(Ac)_2_-Y (10 h).

The morphologies of the adsorbent by SEM are illustrated in Figure 2. With a suitable ion exchange time (10 h), the ZnY crystal particle distribution was uniform, and there was no occurrence of the phenomena of powder and aggregation (Figure 2b–d). 

**Figure 3:**
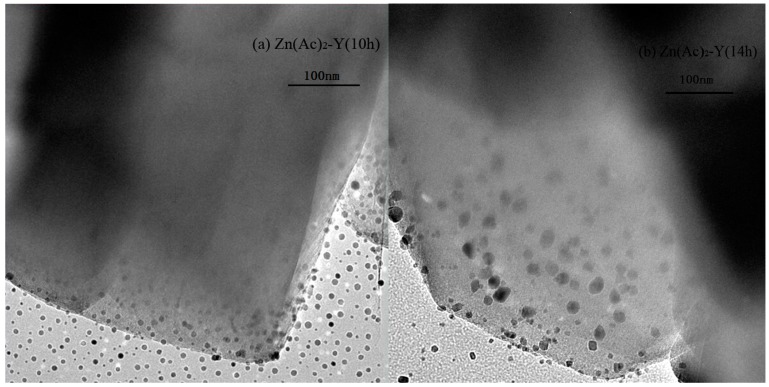
TEM images of adsorbents, (**a**) Zn(Ac)_2_-Y (10 h); (**b**) Zn(Ac)_2_-Y (14 h).

To compare the Zn particle distributions over NaY, TEM micrographs of the samples are presented in Figure 3. The result shows that the Zn particles were dispersed well and confined to the channels of NaY, and large metallic Zn particles are not observed in the Figure 3a. In contrast, in Figure 3b, the ion-exchange time was 14 h, and the Zn species (dark dot-like objects in Figure 3b) in the channels were prone to aggregate and agglomerate on the outer surface of NaY.

### 2.2. Effect of Adsorbent Preparation Conditions

**Figure 4:**
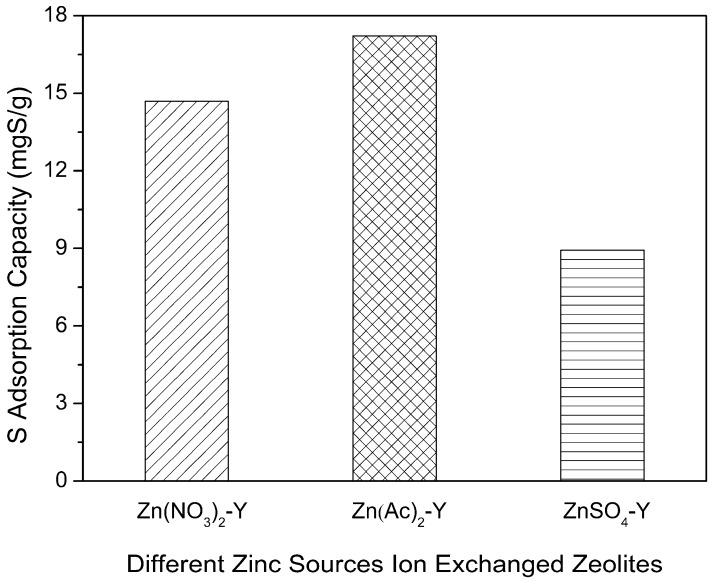
Effect of Zn sources on sulfur adsorption capacity (initial sulfur concentration: 1000 ppmw, T = 40 °C, t = 2 h, adsorbent dosage = 20 g/L; ion-exchange time: 10 h, ion-exchange temperature: 60 °C).

To obtain Zn-exchanged NaY samples with different Zn exchange degrees, three types of Zn sources were employed. The adsorptive desulfurization results are displayed in Figure 4. Notably, Zn(Ac)_2_-Y (10 h, 60 °C) showed the highest sulfur adsorption capacity of 17.21 mg of S/g. The samples of the Zn(NO_3_)_2_-Y (10 h, 60 °C) and ZnSO_4_-Y (10 h, 60 °C) adsorbents had sulfur adsorption capacities of 14.68 and 8.93 mgS/g. Further, the sulfur removals (*R*%) of Zn(Ac)_2_-Y, Zn(NO_3_)_2_-Y and ZnSO_4_-Y (10 h, 60 °C) were 44.21%, 39.58% and 20.29%, respectively. The best performance was observed with the Zn(Ac)_2_-Y (10 h, 60 °C) adsorbent owing to it having the highest Zn exchange degree (59.47%) and the most adsorption activity sites. To elaborate, a higher Zn exchange degree (%) is favorable for a higher sulfur adsorption capacity.

**Figure 5:**
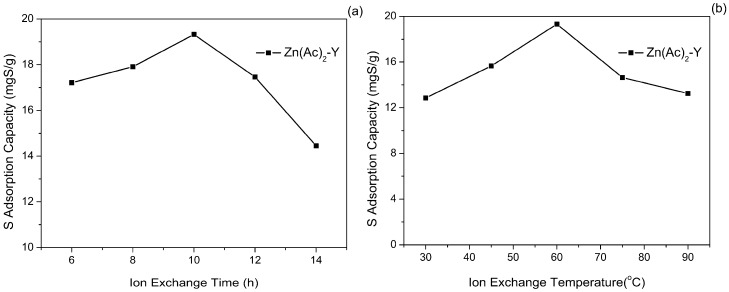
Effect of (**a**) ion-exchange time and (**b**) ion-exchange temperature on sulfur adsorption capacity (initial sulfur concentration: 1000 ppmw, T = 40 °C, t = 2 h, adsorbent dosage = 20 g/L).

The effect of the treatment time of the ion-exchange time on the sulfur adsorption activity by using model gasoline FM-1 at 40 °C is shown in Figure 5a. The sulfur adsorption capacity increased primarily from 17.21 to 19.32 mgS/g when the Zn^2+^ ion-exchange time increased from 6 h to 10 h, and it then dropped quickly to 14.45 mgS/g. As the ion-exchange time prolonged to 14 h, the sulfur adsorption capacity (19.32 mgS/g) and the sulfur removal (52.09%) reached the maximum values. When the ion-exchange equilibrium is reached, increasing the Zn^2+^ ion-exchange time may cause Zn^2+^ clusters to aggregate and agglomerate on the outer surface of NaY, thus gradually decreasing the sulfur adsorption capacity.

The effect of the ion-exchange temperature on the sulfur adsorption capacity was also studied and the results are shown in Figure 5b. It is shown that the sulfur adsorption capacity apparently increased and then decreased with the increase of the ion-exchange temperature, and reached a maximum (19.32 mgS/g) at 60 °C. The content of Zn^2+^ increased as the temperature increased. The increase of the ion-exchange temperature promotes the Na^+^ to get enough energy from the outside to break away from the framework force and form the cation vacancies. Meanwhile, the diffusion rate of Zn^2+^ is accelerates to enter the framework cation vacancies to compensate for the charge. However, further increasing the temperature from 60 to 90 °C caused the Zn^2+^ exchange rate to rise too fast, and this may cause pore blockage, which is detrimental to the sulfur adsorption capacity.

### 2.3. Effect of Adsorption Conditions

**Figure 6:**
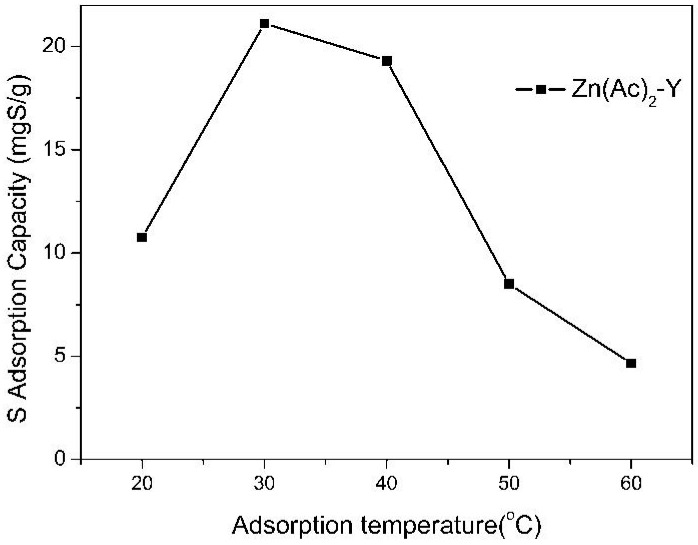
Effect of adsorption temperature on sulfur adsorption capacity (initial sulfur concentration: 1000 ppmw, t = 2 h, adsorbent dosage = 20 g/L; ion-exchange time: 10 h, ion-exchange temperature: 60 °C).

The effect of the adsorption temperature on the sulfur adsorption capacity was investigated in the range of 20 to 60 °C over Zn(Ac)_2_-Y zeolites (10 h, 60 °C), and the results are presented in Figure 6. It was observed that the sulfur adsorption capacity (21.12 mgS/g) and the sulfur removal (56.01%) apparently increased and then decreased with the increase in the temperature and reached a maximum at 30 °C. The reason may be that increasing the system temperature will greatly increase the diffusion rate of the TP, owing to the decrease in the viscosity of the solution. However, when the temperature is >50 °C, the desorption rate will increase sharply, which causes the sulfur adsorption capacity to decrease [41,42,43].

**Figure 7:**
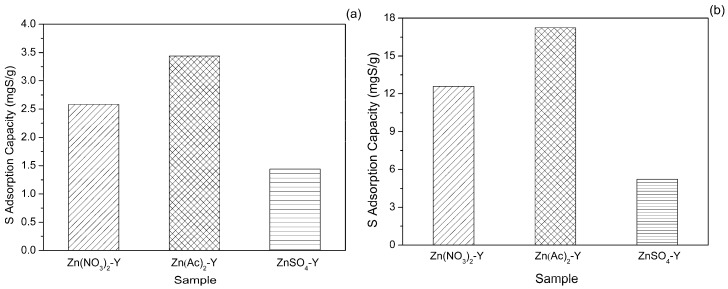
Effect of (**a**) aromatic and (**b**) 1-hexene on sulfur adsorption capacity (initial sulfur concentration: 1000 ppmw, t = 2 h, T = 30 °C, adsorbent dosage = 20 g/L; ion-exchange time: 10 h, ion-exchange temperature: 60 °C).

Gasoline is a complex mixture of hydrocarbons composed mainly of paraffins and aromatics. TP should have a great affinity toward toluene because both are similar species and, as a result, will compete against the zeolite adsorbents for interactions. To clarify the influence of co-existing toluene on TP adsorption, the adsorptive removal of TP on Zn(NO_3_)_2_-Y, Zn(Ac)_2_-Y, ZnSO_4_-Y (10 h, 60 °C) by using MF-2 was investigated. Figure 7a depicts that the desulfurization performance of Zn(Ac)_2_-Y (10 h, 60 °C) was significantly higher than that of Zn(NO_3_)_2_-Y (10 h, 60 °C) and ZnSO_4_-Y (10 h, 60 °C). It also can be seen that in the case of 5 wt % toluene, the sulfur adsorption capacity decreased from 21.12 mgS/g without toluene to 3.44 mgS/g on Zn(Ac)_2_-Y (10 h, 60 °C), which means that the sulfur adsorption capacity declined 83.71%. The results demonstrated that strong competitive adsorption takes place between TP and toluene. Zn^2+^ has the electronic configuration 3d^10^4s^0^. It indicates that there is some donation of the electron charge transfer from the π orbitals of TP to the vacant *s* orbitals of Zn^2+^, simultaneously with the back-donation of the electron charge transfer from the *d* orbitals of Zn^2+^ to the π* orbitals of TP. The toluene contains a benzene ring structure, which forms the competitive adsorption with TP, leading to a sharp drop in the sulfur adsorption capacity [12,31].

In addition to the aromatics, the olefins in the gasoline fuel also have a negative effect on TP removal. To test the influence of co-existing olefin on TP adsorption, the experiments were carried out using Zn(NO_3_)_2_-Y, Zn(Ac)_2_-Y, and ZnSO_4_-Y (10 h, 60 °C) as the adsorbents. It can be seen from Figure 7b that the sulfur adsorption capacity decreased from 21.12 to 17.23 mgS/g. For the samples of ZnSO_4_-Y, the Na exchange degree was 43.17%. Upon ion exchange, ZnSO_4_-Y had the lowest adsorptive active sites. When the adsorption experiments were carried out, the surface of the adsorbents could be clearly observed to generate a little light pink substance. This may be due to the alkylation reaction between olefin and TP, which generates a complex mixture of bulky alkylthiophenes [43]. At the same time, the alkylthiophenes adsorb on the surface active sites of the adsorbent, and block the pores of the adsorbent. Thus, it will restrict TP from entering the super cage and cause the sulfur adsorption capacity to decrease.

### 2.4. Adsorbent Regeneration

**Figure 8:**
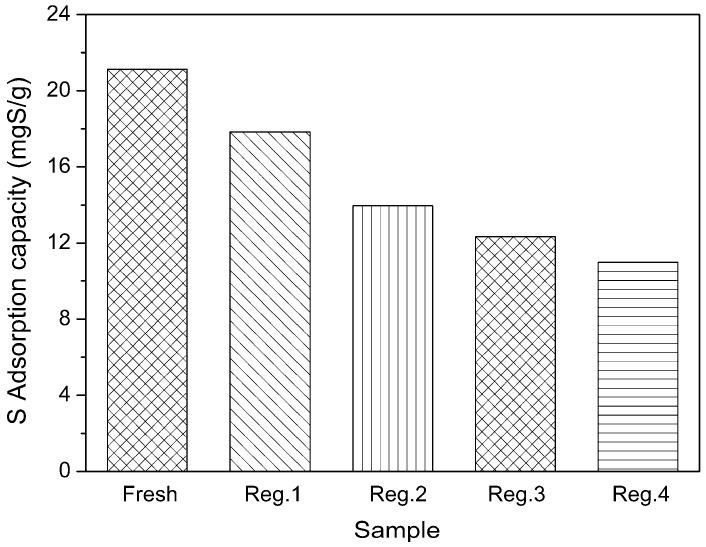
Regeneration performance of Zn(Ac)_2_-Y by thermal treatment (initial sulfur concentration: 1000 ppmw, t = 2 h, T = 30 °C, adsorbent dosage = 20 g/L; ion-exchange time: 10 h, ion-exchange temperature: 60 °C).

**Figure 9:**
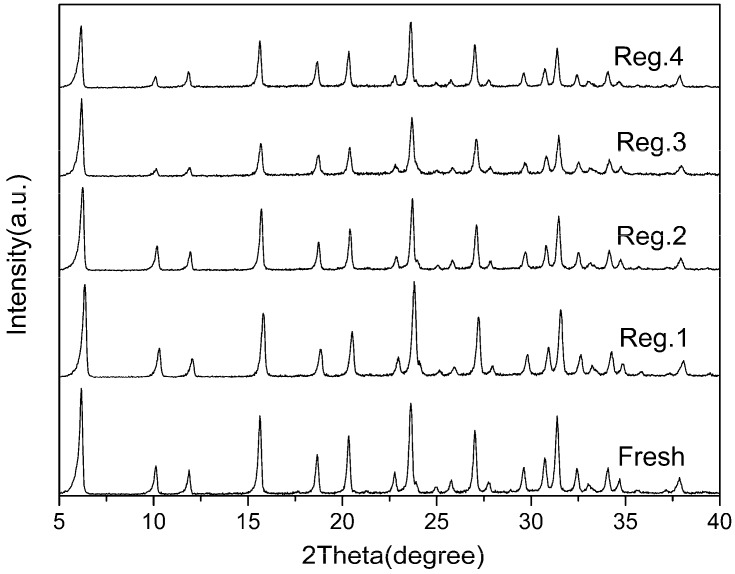
XRD patterns of fresh and regenerated Zn(Ac)_2_-Y.

As illustrated in Figure 8, the adsorbent regenerated by the thermal treatment afforded 84.42%, 66.10%, 58.37% and 51.97% of the initial adsorption capacity for the second, third, fourth and fifth adsorption runs. The sulfur adsorption capacity decreased with the increase of the adsorption-regeneration cycle. This could be due to strong interactions of the Zn^2+^ with the adsorbed TP or, more likely, the low zeolite framework stability of the Zn^2+^. The X-ray diffraction analysis of regenerated Zn(Ac)_2_-Y (10 h, 60 °C) is shown in Figure 9 which indicates that parts of the zeolites’ structure experience crystal lattice collapse and lost crystallinity after the calcination processes.

## 3. Experimental Section

### 3.1. Materials and Adsorbent Spreparation

NaY zeolites (Si/Al = 2.341) in powder form was purchased from Catalyst Plant of Nankai University. TP was obtained from Tianjin Guangfu Chemicals (Tianjin, China). Cyclohexane was purchased from Tianjin Yuanli Chemicals. Toluene was purchased from Tianjin Jiangtian Chemicals (Tianjin, China). The 1-hexene was purchased from Aladdin Chemical Reagent Co., Ltd. (Shanghai, China). Zn(NO_3_)_2_·6H_2_O, Zn(Ac)_2_·2H_2_O, ZnSO_4_·7H_2_O and HAc were commercial reagents in analytical grade and used as received.

Ion-exchange method was used for the preparation of Zn modified Y zeolites. First, 5 g NaY zeolites were treated with 100 mL of 0.1 mol Zn(NO_3_)_2_, ZnSO_4_ and Zn(Ac)_2_ solution, respectively. As Zn(Ac)_2_ is easily to hydrolysis in aqueous solution, therefore, 0.2 mL HAc was added to prevent hydrolysis. Following that, the ion-exchanged zeolites were filtered, washed thoroughly with deionized water to constant pH, and subsequently drying at 100 °C for 6 h. Finally, the samples were calcined at 500 °C for 6 h in air atmosphere. The obtained samples were denoted as Zn(Ac)_2_-Y, Zn(NO_3_)_2_-Y and ZnSO_4_-Y according to different Zn sources.

Model gasoline fuels were prepared by dissolving appropriate amounts of TP into cyclohexane and denoted as MF-1. To obtain more information about S adsorption capacity, 5 wt % of toluene and 5 wt % of 1-hexene were also added and labeled as MF-2 and MF-3, respectively.

### 3.2. Characterizations

The chemical composition of all samples was measured by ICP-AES with a Perkin-Elmer Spectrometer. X-ray diffraction (XRD) patterns of the samples were recorded on a MiniFlex600 X-ray diffract meter (Tokyo, Japan) with Cu-Kβ radiation (λ = 1.54056 Å) operating at 40 KV, 30 mA. Data were collected in the range of 2θ = 5°–40° at a scanning speed of 4°/min. The morphologies of the adsorbents were characterized by SEM (S-4800, Hitachi, Tokyo, Japan) with applied potential of 5 kV. Transmission electron micrographs of the samples were recorded by a JEOL JEM-2100F transmission electron microscope (Tokyo, Japan) with an acceleration voltage of 200 kV.

### 3.3. Adsorption Experiments

The desulfurization from the fuels was conducted by a static adsorption experiments operated at ambient temperature and pressure. Before adsorption, zeolite adsorbents were degassed 6 h at 120 °C. In a typical run, both 25 mL of the model fuel and 0.5 g of the adsorbents were added to a Three-necked flask. The mixtures were treated for 2 h under stirring, afterwards the solution was separated from the adsorbents with a syringe filter. And all the samples collected during the experiments were analyzed using a GC (GC-6820, Santa Clara, CA, USA) equipped with an capillary column and a flame ionization detector (FID).

The sulfur adsorption capacity (*q_t_*) is calculated using the following equation:

(1) $$q_{t}=\frac{\left(c_{0}-c_{t}\right)V\rho }{1000m}$$

The sulfur removal (*R*%) is calculated according to the formula:

(2) $$R\left(\%\right)=\frac{\left(c_{0}-c_{t}\right)}{c_{0}}$$

where *c*_0_ and *c_t_* (ppmw) are the initial and *t* (min) concentration of sulfur in the model fuels, *V* (mL) is the volume of the model fuels, ρ (g/mL) is the density of the model fuels, and *m* (g) is the mass of adsorbent, respectively.

### 3.4. Regeneration Experiments

Safety, high efficiency and availability need to be considered for the regeneration of saturated adsorbents. Heating the spent adsorbents using air is an effective choice for the regeneration of saturated adsorbents. In this work, the feasibility for regeneration of the zeolite adsorbents were investigated by heating the spent zeolite adsorbents. The saturated sample (1.0 g) by MF-1 was dried at 110 °C for 6 h and then calcined at 500 °C for 6 h. To evaluate the reusability of adsorbents, the experiments were carried out for three times at the same conditions.

## 4. Conclusions

A detailed investigation on various Zn^2+^ ion-exchanged NaY zeolite adsorbents confirmed their high capacity for sulfur removal. The sulfur adsorption capacity of ZnY zeolites is dependent on the Zn sources, ion-exchange time and temperature, adsorption temperature and whether they contains aromatics and olefins. A sample of Zn(Ac)_2_-Y, prepared by an ion-exchange method with Zn(Ac)_2_·2H_2_O as a precursor, exhibited the highest capacity for sulfur removal. The excellent performance was related to the higher exchange degree which is controlled by Zn sources. Additionally, there is a suitable temperature and ion-exchange time for the synthesis process.

On the other hand, for the aromatics and olefins that exist (the experiment with Zn(Ac)_2_-Y (10 h, 60 °C)) in the model gasoline, the sulfur adsorption capacity will drop. It is proposed that the Zn^2+^ of Zn(Ac)_2_-Y (10 h, 60 °C) mainly adsorbs TP by π-complexation when the model gasoline fuel contains toluene. The competitive adsorption of toluene and TP has an obvious negative effect on the sulfur adsorption capacity. The 1-hexene in the model gasoline fuel is detrimental to TP removal. This may be due to the alkylation reaction between 1-hexene and TP, which generates a complex mixture of bulky alkylthiophenes that occupy active sites of the adsorbents.

Thermal treatment was used to regenerate the Zn(Ac)_2_-Y (10 h, 60 °C) adsorbent spent by MF-1, and the regenerated Zn(Ac)_2_-Y (10 h, 60 °C) samples were characterized. The X-ray diffraction analyses indicated that parts of the zeolites’ structure experienced crystal lattice collapse and lost crystallinity after the calcination processes. The sulfur adsorption capacity decreases with the increase of the adsorption-regeneration cycle. This could be due to the low zeolite framework stability of the Zn cations.

## Figures and Tables

**Table 1:** Chemical content of the samples prepared under different conditions.

Sample	Preparation Condition	Chemical Content (mol/g)	Molar Ratio	Na Exchange Degree (%)
	Ion Exchange Time (h)	Zn	Si/Al	
NaY	-	-	2.341	-
Zn(NO_3_)_2_-Y	10	0.894	2.346	56.70
ZnSO_4_-Y	10	0.702	2.384	43.17
Zn(Ac)_2_-Y	6	0.672	2.342	39.62
	10	0.969	2.386	59.47
	14	1.206	2.391	82.82
